# A putative lipase affects *Pseudomonas aeruginosa* biofilm matrix production

**DOI:** 10.1128/msphere.00374-23

**Published:** 2023-09-27

**Authors:** Somalisa Pan, Mary Erdmann, Julia Terrell, Matthew T. Cabeen

**Affiliations:** 1 Department of Microbiology and Molecular Genetics, Oklahoma State University, Stillwater, Oklahoma, USA; University of Kentucky College of Medicine, Lexington, Kentucky, USA

**Keywords:** phospholipase, biofilms, *Pseudomonas aeruginosa*, matrix, *bipL*, lipidomics

## Abstract

*Pseudomonas aeruginosa* is an opportunistic pathogen that is widely known for infecting patients with underlying conditions. This species often survives antibiotic therapy by forming biofilms, in which the cells produce a protective extracellular matrix. *P. aeruginosa* also produces virulence factors that enhance its ability to cause disease. One signaling pathway that influences virulence is the nitrogen-related phosphotransferase system (Nitro-PTS), which consists of an initial phosphotransferase, *PtsP*, a phosphocarrier, PtsO, and a terminal phosphate receptor, PtsN. The physiological role of the Nitro-PTS in *P. aeruginosa* is poorly understood. However, PtsN, when deprived of its upstream phosphotransfer proteins, has an antagonistic effect on biofilm formation. We thus conducted a transposon mutagenesis screen in an unphosphorylated-PtsN (i.e., ∆*ptsP*) background to identify downstream proteins with unacknowledged roles in PtsN-mediated biofilm suppression. We found an unstudied gene, *PA14_04030*, whose disruption restored biofilm production. This gene encodes a predicted phospholipase with signature alpha/beta hydrolase folds and a lipase signature motif with an active-site Ser residue. Hence, we renamed the gene *bipL*, for biofilm-impacting phospholipase. Deletion of *bipL* in a ∆*ptsP* background increased biofilm formation, supporting the idea that BipL is responsible for reducing biofilm formation in strains with unphosphorylated PtsN. Moreover, substituting the putative catalytic Ser for Ala phenocopied *bipL* deletion, indicating that this residue is important for the biofilm-suppressive activity of BipL *in vivo*. As our preliminary data suggest that BipL is a lipase, we performed lipidomics to detect changes in the lipid profile due to *bipL* deletion and found changes in some lipid species.

**IMPORTANCE:**

Biofilm formation by bacteria occurs when cells secrete an extracellular matrix that holds them together and shields them from environmental insults. Biofilms of bacterial opportunistic human pathogens such as *Pseudomonas aeruginosa* pose a substantial challenge to clinical antimicrobial therapy. Hence, a more complete knowledge about the bacterial factors that influence and regulate production of the biofilm matrix is one key to formulate more effective therapeutic strategies. In this study, we screen for factors that are important for reducing biofilm matrix production in certain genetic backgrounds. We unexpectedly found a gene encoding a putative lipase enzyme and showed that its predicted catalytic site is important for its ability to reduce biofilm formation. Our findings suggest that lipase enzymes have previously uncharacterized functions in biofilm matrix regulation.

## INTRODUCTION

*Pseudomonas aeruginosa* is a Gram-negative opportunistic pathogen that is well known to cause infections in nosocomial settings (1), particularly targeting immunocompromised patients with underlying conditions such as cystic fibrosis (2 – 4) or burn wounds (5 – 7). *P. aeruginosa* exhibits several virulence behaviors that contribute to its pathogenicity (8). Biofilm formation, which is characterized by the production of extracellular polymeric substances (EPS) that hold cells together and act as a protective shield (9 – 11), is one such behavior that helps this pathogen to colonize host tissues and medical devices in hospital settings, making it resistant to antimicrobial therapy and a major public health concern (9, 12, 13).

The nitrogen-related phosphotransferase (Nitro-PTS) system in *P. aeruginosa*, which shares homology with classical sugar phosphotransferase systems (PTS) in bacteria, consists of three components: a kinase, *PtsP* (also known as Enzyme I or EI); a phosphocarrier, PtsO (also known as Npr); and a terminal phosphate receptor, PtsN (also known as Enzyme II or EII) (14). Unlike the EII of a classical sugar PTS, the terminal phosphate receptor PtsN does not phosphorylate imported saccharides but rather has regulatory roles, including on EPS production and type III secretion in *P. aeruginosa* (15, 16) and on potassium transport, cell motility, and other phenotypes in *Escherichia coli* and *Pseudomonas putida* (17 – 19).

Biofilm formation by *P. aeruginosa* can be assessed in a multitude of ways but one facile method is to visually inspect colony morphology. A wrinkled colony appearance typically corresponds to increased EPS production, whereas a smooth colony appearance indicates reduced EPS (15, 20). Colony wrinkling in strain PA14, which we use here, occurs in response to increased Pel polysaccharide production (20). The amount of Pel present in a biofilm colony can be further quantified using the Congo red dye (15, 20, 21). Using these methods, we previously found that strains deleted for *ptsP* or a *ptsO* displayed substantially reduced Pel levels compared to a modestly hyper-biofilm forming Δ*amrZ* parental background (15). As deletion of *ptsP* fully blocks PtsN phosphorylation and *ptsO* deletion partially blocks PtsN phosphorylation (22), this finding suggested that disruption of phosphate flow through the Nitro-PTS reduces Pel production. Surprisingly, deletion of *ptsN* restored biofilm formation, implicating unphosphorylated PtsN as a factor in suppressing Pel production (15). The mechanism by which PtsN impacts Pel production remains unknown.

Here, we conducted a transposon mutagenesis screen using a strain background (Δ*amrZ* Δ*ptsP*) in which the Nitro-PTS suppresses Pel production (15). We sought to identify genes whose disruption would restore Pel production, with the idea that the products of such genes might be involved in Nitro-PTS-mediated biofilm suppression. We found an unnamed gene, *PA14_04030*, whose disruption resulted in restoration of wrinkled colony morphology and Pel production. Unexpectedly, we found that the 04030 protein shares homology with alpha-beta hydrolase folds common to lipase enzymes. Here, we identify the putative active-site serine of this lipase and examine its importance in Pel production. We also show alterations to the lipid profile of PA14 in the absence of 04030, corroborating its putative lipase function. We also renamed this gene and protein as *bipL*, for biofilm-impacting phospholipase.

## RESULTS

### Visual screening identifies *bipL* as a factor in the reduced-biofilm phenotype of Δ*amrZ* Δ*ptsP*

**Fig 1:**
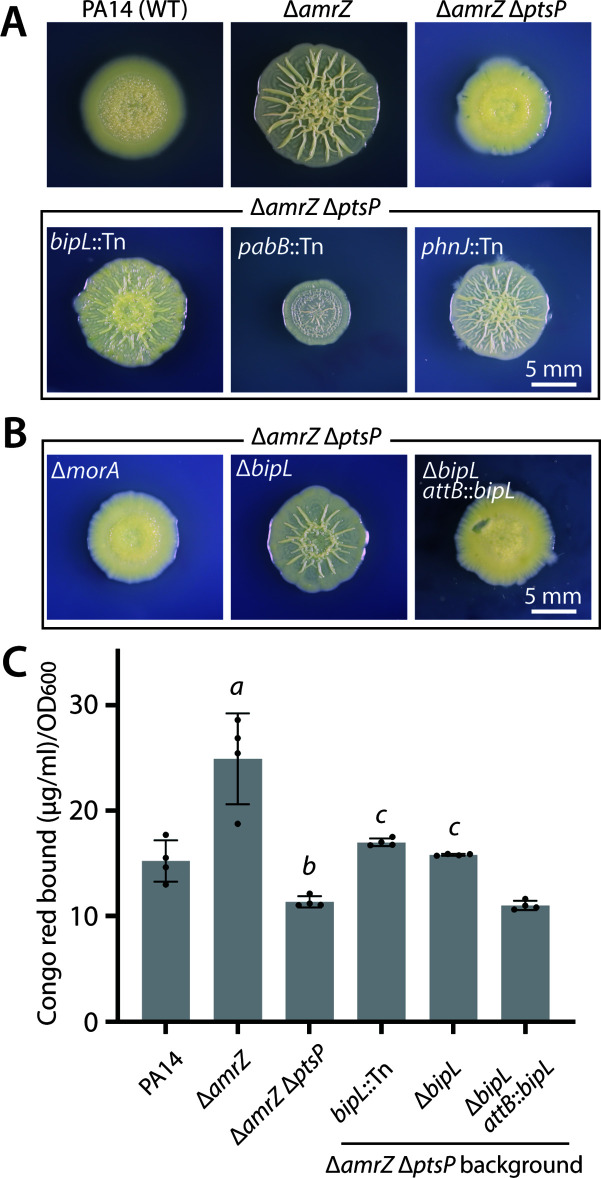
Impacts of *bipL* disruption or deletion on colony morphology and Pel production. (**A and B**) Representative photographs of colony morphology of the indicated strains after 6 days of growth at 25°C on M6301 agar. (**C**) Congo red-binding assay of selected strains from panels A and B. Bar graphs indicate the mean values of four biological replicates whose individual values are also shown as dots. Error bars denote standard deviation. Significance was assessed by two-tailed Student’s *t*-test. *a*, *P* < 0.01 vs PA14; *b*, *P* < 0.001 vs Δ*amrZ*; and *c*, *P* < 0.00001 vs Δ*amrZ* Δ*ptsP*.

We previously reported that deletion of *ptsP*, encoding Enzyme I of the Nitro-PTS, in the moderately hyper-biofilm forming Δ*amrZ* background resulted in clearly decreased colony wrinkling and Pel polysaccharide levels (15). In accord with its reduced production of biofilm matrix, the *ptsP* mutant also displayed reduced intracellular cyclic di-GMP levels (15). Additional deletion of *ptsN*, encoding Enzyme II, restored colony wrinkling, Pel production, and cyclic-di-GMP, implicating PtsN as a negative regulator of biofilm formation (15). As cyclic-di-GMP is one of the key second messengers that drives the biofilm lifestyle of this bacterium (23), we hypothesized that deletion of *ptsP* and the consequent unphosphorylated state of PtsN (16) might somehow activate a downstream phosphodiesterase (PDE) that would cleave cyclic-di-GMP, reducing its intracellular concentration and thus lowering Pel production. To uncover such a hypothetical PDE, we performed a visual screen of transposon-mutagenized ∆*amrZ* ∆*ptsP*, reasoning that disruption of a downstream PDE—or any downstream factor important for reducing Pel levels in the *amrZ* ∆*ptsP* background—would tend to restore Pel production and the consequent wrinkled colony morphology. After screening approximately 750 transposon mutant colonies, we uncovered four with restored wrinkling (Fig. 1A). These mutants harbored transposon insertions in *PA14_04030* (encoding a putative phospholipase), *morA* (encoding a bifunctional diguanylate cyclase-PDE), *pabB* (encoding a para-aminobenzoate synthase gene), and *phnJ* (encoding a hypothetical protein within a phosphonate metabolism cluster). Despite our screen being far from saturated, we suspended the screen to characterize our first four hits. While we were initially excited to find an insertion in *morA* because of its role in cyclic-di-GMP production and breakdown (24 – 26), subsequent deletion of *morA* did not enhance colony morphology (Fig. 1B), leading us to conclude that transposon insertion resulted in production of an aberrantly active diguanylate cyclase. Our initial efforts to delete *pabB* and *phnJ* were unsuccessful, and, intrigued by the predicted function of 04030 as a phospholipase, we focused our efforts on the *PA14_04030* gene (renamed *bipL*). We verified the effect of *bipL* on biofilm formation by constructing a markerless in-frame deletion of *bipL* in an Δ*amrZ* Δ*ptsP* background. Deletion of *bipL* achieved the same restoration of colony wrinkling as the transposon insertion (Fig. 1A and B), thus implicating *bipL* as a mediator of biofilm (Pel) reduction. As a further test, we chromosomally complemented *bipL* under its endogenous promoter at the *attB* locus. The complemented strain displayed smooth morphology that was indistinguishable from the parental Δ*amrZ* Δ*ptsP* (Fig. 1B), firmly establishing *bipL* as a negative regulator of biofilm colony wrinkling in this strain background. In addition to inspecting colony morphology, we also deployed a Congo red-binding assay to quantify the Pel extracellular polysaccharide produced by colonies of each strain. The results of this assay were in strong agreement with the visual morphological changes, showing a significant increase in Pel levels in the absence of *bipL* that was completely reversed by *bipL* complementation (Fig. 1C).

### *bipL* deletion does not universally increase Pel production across different strain backgrounds

**Fig 2:**
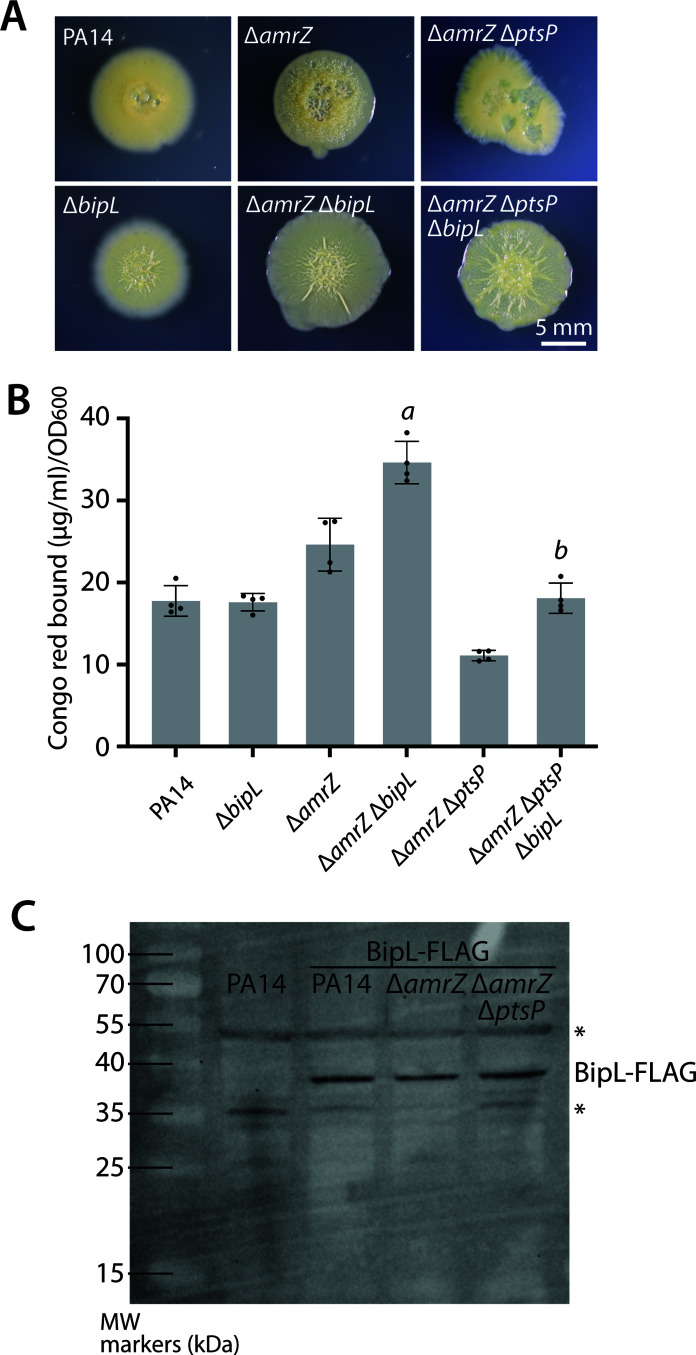
Impacts of *bipL* disruption in different strain backgrounds. (**A**) Representative photographs of colony morphology of the indicated strains after 6 days of growth at 25°C on M6301 agar. (**B**) Congo red-binding assay of the same strains as shown in panel A. Bar graphs indicate the mean values of four biological replicates whose individual values are also shown as dots. Error bars denote standard deviation. Significance was assessed by two-tailed Student’s *t*-test. *a*, *P* < 0.005 vs Δ*amrZ* and *b*, *P* < 0.0005 vs Δ*amrZ* Δ*ptsP*. (C) Anti-FLAG immunoblot of OD_600_-normalized cell lysates from the indicated strains to detect BipL-FLAG produced as the only source of BipL in the cell from the native chromosomal locus. Asterisks indicate nonspecific bands appearing even in PA14 extract (with no FLAG-tagged proteins).

The goal of our visual screen was to find genes that were important for the reduced-biofilm phenotype corresponding to *ptsP* deletion. After unexpectedly identifying *bipL* as such a gene, we next asked whether *bipL* deletion would broadly increase Pel production across all strain backgrounds or whether its effect was narrower—for instance, limited to *ptsP*-deleted cells. To distinguish these possibilities, we constructed markerless, in-frame deletions of *bipL* in a wild-type PA14 background, which is a weak biofilm former, and in an Δ*amrZ* background, which moderately overproduces Pel and appears hyper-wrinkled (15, 27, 28). Deletion of *bipL* affected the visual appearance of colonies in both backgrounds (we also show the Δ*amrZ* Δ*ptsP* background for reference), notably reducing the prominence of spoked wrinkles in the Δ*amrZ* background (Fig. 2A). However, when we assessed Pel levels via Congo red binding, we observed significant increases in Pel production in the Δ*amrZ* and Δ*amrZ* Δ*ptsP* backgrounds but not in the wild-type background (Fig. 2B). Repeated trials of this experiment (Fig. S1) revealed that the most consistent and statistically significant increases in Pel production occurred when *bipL* was deleted from the Δ*amrZ* Δ*ptsP* background. From these results, we conclude that *bipL* deletion does not simply increase Pel production in any strain background but shows its strongest effects in *ptsP*-deleted cells and, to a lesser extent, in the Δ*amrZ* background.

### Neither expression of *bipL* nor abundance of BipL protein is altered by *ptsP* deletion

Because we observed the strongest effect of *bipL* deletion in a Δ*amrZ* Δ*ptsP* background, we considered the possibility that *bipL* might be transcriptionally activated in the cells deleted for *ptsP*. However, when we performed transcriptomic comparisons of the hyper-biofilm Δ*amrZ* strain to the biofilm-suppressed Δ*amrZ*Δ*ptsP* strain under identical colony growth conditions, we observed no differential regulation in the expression of *bipL* (1.02-fold change, *P* = 0.94, Δ*amrZ* Δ*ptsP* vs Δ*amrZ*), arguing against Nitro-PTS-mediated regulation of *bipL* expression. We thus considered the further possibility that *ptsP* deletion might affect the production or stability of the BipL protein. Using a C-terminally FLAG-tagged version of BipL expressed as an allelic replacement from its native locus, we performed anti-FLAG immunoblotting for BipL-FLAG in three strain backgrounds—PA14, Δ*amrZ*, and Δ*amrZ* Δ*ptsP*. We observed very similar BipL levels in each strain background (Fig. 2C), suggesting that neither the absence of AmrZ nor the absence of *PtsP* impacts BipL protein levels.

### BipL is a predicted alpha/beta hydrolase with hallmarks of lipase proteins

Our initial expectation for our transposon screen was that we might find a PDE that cleaved cyclic-di-GMP. Contrary to this expectation, protein analysis tools such as Alphafold (29, 30) and PredictProtein (31) deemed BipL to be a lysophospholipase based on sequence homology. Still, we considered the formal possibility that BipL might have PDE activity, as lysophospholipases are also known to cleave phosphodiester bonds (32), and a human lysophospholipase D called autotaxin also functions as a nucleotide phosphodiesterase (33). Lysophospholipase D enzymes include conserved catalytic HKD (histidine, lysine, and aspartic acid residue) motifs (34). The conserved histidine is separated by seven non-conserved residues from the KxD residues (34). When we examined the amino acid sequence of BipL, we observed the presence of a similar HxxxxxxxKxD motif beginning at residue 268 (Fig. S2A). To test whether these residues were important for the biofilm-impacting function of BipL, we genetically engineered BipL mutants in which the His residue or all three HKD residues were substituted with Ala. We reasoned that if these residues were important for the function of the BipL protein, the substitutions would render BipL non-functional and phenocopy the *bipL* deletion. However, none of the mutants phenocopied the *bipL* deletion with respect to colony morphology (Fig. S2B), suggesting that this conserved motif, even if it has a catalytic function, does not impact biofilm formation.

**Fig 3:**
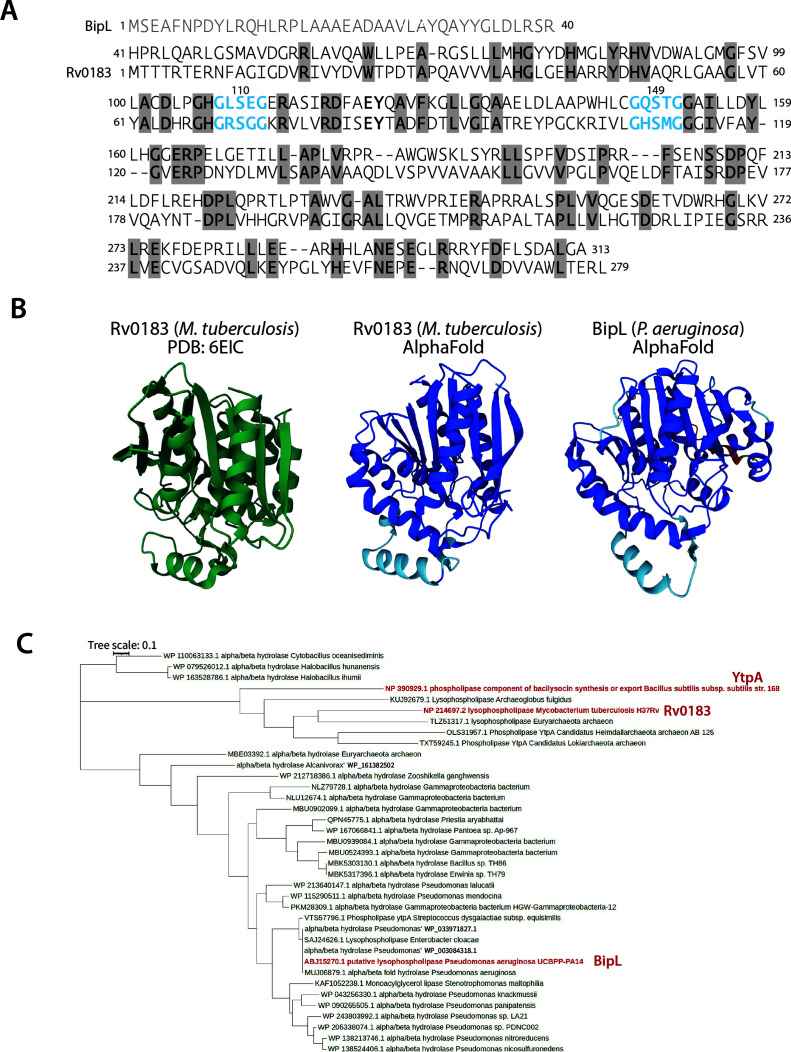
Similarity of BipL to bacterial lipase enzymes. (A) Primary peptide sequence alignment between BipL from *P. aeruginosa* and Rv0183 from *M. tuberculosis*. Amino acid positions are shown at the beginning and end of each line. Areas of identity are shaded in grey, and GXSXG signature lipase motifs are shown in blue. (B) Ribbon diagrams of the reported crystal structure of *M. tuberculosis* Rv0183 (green) and of the Alphafold-predicted structures of Rv0183 and BipL (blue). (C) Phylogenetic tree comparing bacterial alpha/beta hydrolase protein similarity and showing the relative positions of BipL, Rv0183, and a studied *Bacillus subtilis* lipase enzyme (red).

Further examination of BipL revealed that it shares homology with the alpha-beta hydrolase family that includes lipase enzymes. Lipases often bear a conserved catalytic triad consisting of histidine, serine, and aspartic acid, where the catalytic serine is a part of a signature motif that is native to lipase enzymes—the GXSXG motif (35). We identified two such motifs in BipL (Fig. 3A, blue) with two potential catalytic Ser residues at positions 110 and 149. We further used the protein modeling software Alphafold to generate a putative structural model of BipL. Its structure was at least similar to Rv0183 of *Mycobacterium tuberculosis* (Fig. 3B), a protein to which BipL shares sequence homology (Fig. 3A) and which is an experimentally characterized lipase (36). Notably, while the actual crystal structure of Rv1083 (Fig. 3B, green) (37) differs slightly from the Alphafold prediction (Fig. 3B, center structure, blue), the predicted structure of BipL strongly resembles the *predicted* structure of Rv1083 (Fig. 3B, blue), suggesting significant similarities between the two proteins. We also sought insight into the conservation of BipL-like proteins in different organisms. We used Basic Local Alignment Search Tool (BLAST) to identify proteins with homology to BipL and then manually selected homologous sequences from taxonomically diverse organisms to align with Clustal Omega and then construct a phylogenetic tree using MEGA11. The phylogenetic tree shows that BipL-like putative lipase enzymes are common across different bacterial species (Fig. 3C). The tree also includes enzymes with experimentally demonstrated lipase activity, including Rv0183 (36) and the *Bacillus subtilis* enzyme YtpA (38) (Fig. 3C, red).

### The putative catalytic Ser 149 is crucial for BipL-mediated biofilm suppression

**Fig 4:**
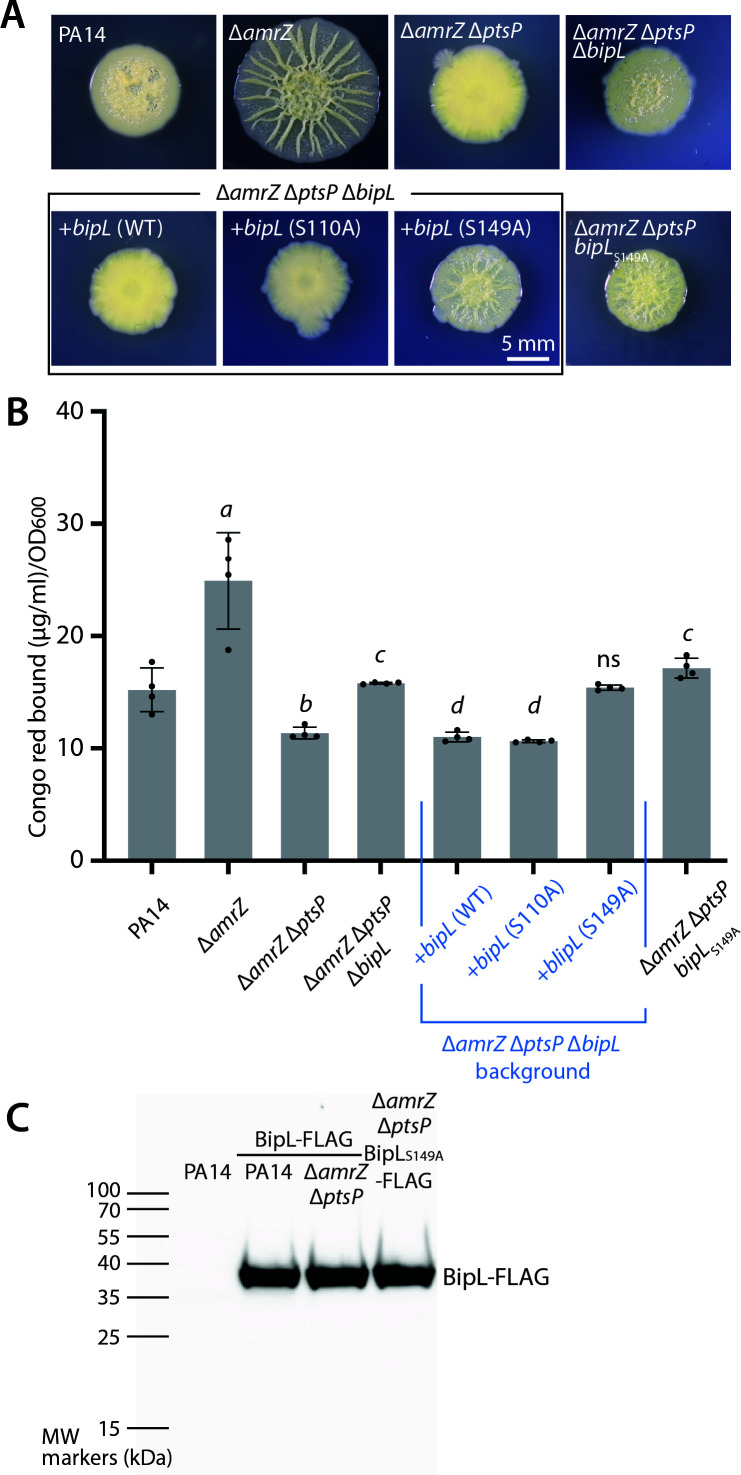
Identification of Ser 149 as a functionally important residue in BipL. (A) Representative photographs of colony morphology of the indicated strains after 6 days of growth at 25°C on M6301 agar. (B) Congo red-binding assay of the same strains as shown in panel A. Bar graphs indicate the mean values of four biological replicates whose individual values are also shown as dots. Error bars denote standard deviation. Significance was assessed by two-tailed Student’s *t*-test. *a*, *P* < 0.01 vs PA14; *b*, *P* < 0.001 vs Δ*amrZ*; *c*, *P* < 0.00005 vs Δ*amrZ* Δ*ptsP; d*, *P* < 1 x 10^−6^ vs Δ*amrZ* Δ*ptsP* Δ*bipL*; and ns, *P* > 0.02 vs Δ*amrZ* Δ*ptsP* Δ*bipL*. (C) Anti-FLAG immunoblot of OD_600_-normalized cell lysates from the indicated strains to detect BipL-FLAG or BipL_S149A_-FLAG produced as the only source of BipL in the cell from the native chromosomal locus.

Given the strong prediction that BipL is a lipase enzyme, we next asked whether mutation of the putative active-site Ser residues would affect the biofilm-modulating function of BipL. We therefore constructed individual Ala substitutions of the two putative catalytic Ser residues in the two identified GXSXG motifs (Fig. 3A, blue) at positions 110 and 149 to learn whether either is important for BipL-mediated biofilm suppression. We took a complementation strategy, expressing each Ser-to-Ala substituted version of BipL under the endogenous promoter from an ectopic locus (*attB*) in an Δ*amrZ* Δ*ptsP* Δ*bipL* background. We then visually assessed the ability of each version of BipL to shift the morphology of the Δ*amrZ* Δ*ptsP* Δ*bipL* strain from wrinkled to smooth. Such a shift, phenocopying the wild-type *bipL* complementation and reducing Pel production, would indicate that the mutated Ser residue is unimportant for the ability of BipL to lower Pel production. Conversely, a failure to shift to a smooth morphology would indicate abrogation of BipL function with respect to Pel regulation. Expression of *bipL*_S110A_ yielded a smooth phenotype that was indistinguishable from the wild-type complementation, both visually (Fig. 4A) and when Pel was quantitated by Congo red binding (Fig. 4B). In sharp contrast, expression of *bipL*_S149A_ failed to reverse the wrinkled morphology of the parental strain, indicating that Ser 149 has a critical role in the ability of BipL to mediate reduced Pel levels (Fig. 4A and B). As a secondary test of the function of Ser 149, we expressed *bipL*_S149A_ as an allelic replacement from the native chromosomal locus in a Δ*amrZ* Δ*ptsP* background. Supporting the result using the complementation strategy, the S149A substitution showed wrinkled morphology and elevated Pel levels (Fig. 4A and B).

To rule out the possibility that the S149A substitution impacted BipL stability or abundance, we replaced native *bipL* with *bipL*_S149A_-*flag* in a Δ*amrZ* Δ*ptsP* background and used anti-FLAG blotting to assess the abundance of the S149A mutant relative to the wild type. We observed no discernable difference between the wild-type and mutant protein (Fig. 4C), suggesting that the S149A substitution does not impact protein stability or abundance. Moreover, neither Ser-to-Ala substitutions at serines 50, 98, or 116 nor Asp-to-Ala substitutions at aspartates 197 or 210 phenocopied *bipL* deletion as the S149A substitution did (Fig. S3). We infer from these collective data that Ser 149 has an important and specific role in the ability of BipL to suppress Pel production in Δ*amrZ* Δ*ptsP* cells.

### Lipidomic analysis shows alterations in PA14 polar lipids in the absence of BipL

**Fig 5:**
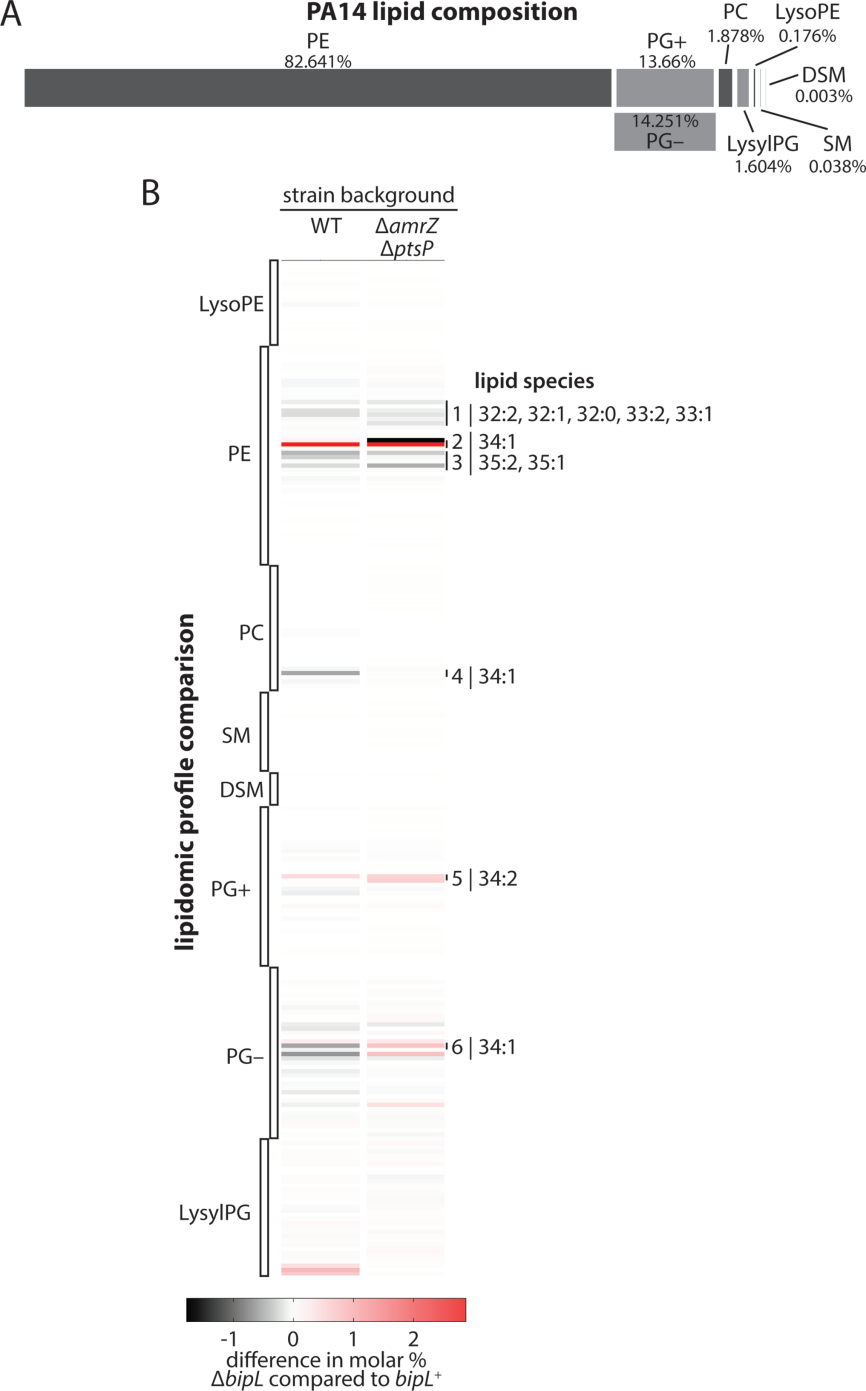
Lipid profiles of strains deleted and not deleted for *bipL*. (**A**) Overall phospholipid composition of wild-type PA14 cells. PE, phosphatidylethanolamine; PG, phosphatidylglycerol (the + and – indicate PG detected in positive- and negative-ion mode, respectively); PC, phosphatidylcholine; LysylPG, lysylphosphatidylglycerol; LysoPE, lysophosphatidylethanolamine; SM, sphingomyelin; DSM, dihydrosphingomyelin. (**B**) Heatmap highlighting relative differences in abundance (in Δ*bipL* vs *bipL* ^+^ cells in the indicated strain backgrounds) of the indicated lipid classes and species. The numbers given for lipid species (e.g., 34:1) denote the total number of carbons in the fatty acid tails and the number of unsaturations, respectively.

Given the strong resemblance of BipL to lipase enzymes, we next sought to experimentally verify whether BipL has lipase activity. Our initial strategy was to purify BipL and perform a standard lipase assay. We purified His-tagged BipL and used the purified protein to conduct a lipase assay using commercially available 4-nitrophenyl laurate, with the expectation that a lipase may cleave a *p*-nitrophenyl ester to release *p*-nitrophenol, which will increase OD_410_ (39). However, we did not detect any lipolytic activity (data not shown). We considered many explanations for this negative result, including the possibility that our *in vitro* conditions did not accurately replicate *in vivo* conditions and that BipL has no activity against the lauryl (C_12_) substrate we used. To circumvent these uncertainties, we instead turned to lipidomic analysis to determine how the presence or absence of BipL impacts the total lipid profile of *P. aeruginosa* PA14. We extracted lipids from strains deleted or not for *bipL* in both wild-type PA14 and Δ*amrZ* Δ*ptsP* backgrounds (four strains in total). The lipidomic analysis revealed specific changes in the lipid profile of *P. aeruginosa* when *bipL* was deleted. The bulk of the identified lipids in our samples were phosphatidylethanolamines (PE), with phosphatidylglycerol (PG) representing another substantial fraction (Fig. 5A). These results are overall consistent with previous studies of *P. aeruginosa* phospholipid content (40, 41). Other lipid species, including phosphatidylcholines (PC), sphingomyelin (SM), lysylphosphatidylglycerol (LysylPG), lysophosphatidylethanolamine (LysoPE), and dihydrosphingomyelin (DSM), were detected but in very small relative quantities (Fig. 5A). Most of the changes detected upon *bipL* deletion were observed among phosphatidylethanolamines and phosphatidylcholines, with some lipid species showing greater abundance and others showing reduced abundance (a full list of each species detected in each sample and the differences among them are given in Data S1). One pattern that emerged among the most-changed lipid species was phospholipids with 34 total carbons in the fatty acid tails (Fig. 5B). These results, while not definitive, at least hint at a role for BipL in the metabolism of phospholipids with, for example, 16- and 18-carbon fatty acid tails, which constitute the bulk of detected fatty acids in *P. aeruginosa* (42, 43), particularly in biofilm-derived samples (43). While it remains unclear at present how this potential lipase activity of BipL is related to its observed role in Pel production, future work may establish a connection between such lipid composition changes and biofilm behavior in *P. aeruginosa*.

## DISCUSSION

In this study, we report a previously unappreciated role for a putative lipase in *P. aeruginosa* in biofilm regulation. With the motive of exploring downstream targets of the Nitro-PTS system, which potentially act as biofilm regulators, we conducted transposon mutagenesis screening in the Δ*amrZ* Δ*ptsP* background, which has a reduced-biofilm phenotype. An initial screen of approximately 750 transposon mutants identified four insertions with restored biofilm levels (Fig. 1A). As the screen is far from saturated, and as we found only one mutant with insertion in a known PDE (44), there may be other proteins downstream of the Nitro-PTS that remain to be discovered. However, in our initial screen, we uncovered the previously uncharacterized gene *PA14_04030* (renamed here *bipL*), whose loss restores Pel production and colony wrinkling. Importantly, these phenotypes argue that the reductions in Pel levels seen in *ptsP-*deleted strains depend at least in part on the activity of the BipL protein, implicating BipL as a negative downstream effector of biofilm formation. However, as *bipL* deletion did not increase Pel levels in colony biofilms formed by wild-type cells, we conclude that the effect of BipL is limited to certain strain backgrounds, including Δ*amrZ* Δ*ptsP* and, to a lesser extent, Δ*amrZ*. Transcription of *bipL* was similar between these backgrounds and between Δ*amrZ* and the wild type (fold change 1.37, *P* = 0.25, Δ*amrZ* vs PA14), suggesting that *bipL* expression is not induced in the strains in which it has the greatest biofilm effect. Similarly, the overall levels of BipL protein were similar in different strain backgrounds, again arguing that BipL activity is not regulated by protein abundance. However, none of our results rule out the possibility that the effect of BipL on Pel levels is controlled post-translationally, perhaps via interactions among proteins.

Intriguingly, a predicted GXSXG lipase active-site motif of BipL centered on Ser 149, one of two in its peptide sequence, was important for its biofilm-impacting function. While we were unable, in the present work, to experimentally establish lipase activity for BipL *in vitro*, these results are at least suggestive of a link between the enzymatic activity of BipL and its impact on biofilm formation. The similarity of the structural prediction of BipL to that of an experimentally verified bacterial lipase (Fig. 3B) constitutes another line of evidence suggesting that BipL is a lipase. Indeed, the lipidomic approach we took to detect any lipolytic activity of BipL showed detectable changes in lipid species in the absence of *bipL*, at least consistent with the lipase function for BipL. Moreover, phospholipids with a total of 34 carbons (e.g., 34:1 and 34:2) were conspicuous among the changed species. As phospholipids have two fatty acid tails, 34:1 and 34:2 phospholipids correspond to the 16:0, 16:1, and 18:1 fatty acids that compose the bulk of detected fatty acids in *P. aeruginosa* biofilms (42, 43). The mechanism by which lipase activity of BipL and alterations to 34-carbon phospholipid content may impact Pel production and biofilm formation remains unknown at present; however, this study is one of the first to connect a lipase enzyme to bacterial biofilm formation.

A previous study (45) reported a link between lipase, LipC, and biofilm formation in *P. aeruginosa*, finding that altered rhamnolipid production and cell motility in a *lipC* mutant altered the biofilm architecture of the bacteria such that surface coverage by biofilm-forming cells was enhanced. Such a phenotype is consistent with the enhancement of biofilm matrix production by the loss of *bipL*. However, the biofilm-forming ability of certain Gram-positive *Staphylococcus aureus* strains appears to be enhanced in the presence of lipases (46). Such a finding contrasts with our study, in which the absence of the lipase or abrogation of its active-site Ser corresponds to increased Pel production and enhanced biofilm formation. Our finding warrants additional work to learn whether BipL truly has lipase activity and how its enzymatic activity, whatever it be, influences Pel production. It is also possible that BipL is not a lipase or, even if it does have lipase activity, that such activity is distinct from its role in biofilm regulation. It also remains to be discovered how BipL may interact with or be signaled to by the Nitro-PTS and by PtsN specifically. In any case, the identification of enzymes that can act to negatively regulate biofilm matrix production represents an exciting prospect for developing future anti-biofilm therapeutic strategies.

## MATERIALS AND METHODS

### Strains and growth conditions

*P. aeruginosa* PA14 and *E. coli* SM10 were grown overnight in Luria Bertani (LB)-Lennox (10 g/L tryptone, 5 g/L yeast extract, 5 g/L NaCl) media. The liquid cultures were grown in a 37°C shaking incubator. To select strains with specific antibiotic cassette insertions, antibiotics (75  µg/mL or 20  µg/mL gentamicin, 25  µg/mL tetracycline, or 50 µg/mL kanamycin) were added to the media as required. To generate markerless in-frame deletions or point mutations, PA14 cells were mated with SM10 cells with a pEXG2 vector, followed by selection against *E. coli* cells by adding 25 µg/mL Irgasan to the medium. The strains were further counterselected on LB plates containing 6% sucrose. Deletions and point mutations were confirmed by screening PCRs followed by sequencing. Complemented strains were constructed using mini-CTX plasmid, which integrates at the chromosomal *attB* locus. The strains used in this study are listed in Table 1. The plasmids and primers used to construct all the strains are listed in the supplemental material.

**TABLE 1:** *Pseudomonas aeruginosa* strains used in this study

Strain	Genotype or description	Source or reference
MTC1	Laboratory wild-type strain of *P. aeruginosa* (PA14)	Laboratory stock; Stephen Lory, Harvard Medical School
CSS195	PA14 Δ*amrZ* Δ*ptsP pabB*::*Tn*	This study
CSS198	PA14 Δ*amrZ* Δ*ptsP bipL*::*Tn*	This study
CSS205	PA14 Δ*amrZ* Δ*ptsP pabB*::*Tn*	This study
MTC590	PA14 Δ*amrZ*	(15)
MTC1387	PA14 Δ*amrZ* Δ*ptsP*	(15)
MTC2032	PA14 Δ*amrZ* Δ*ptsP* Δ*morA*	This study
MTC2068	PA14 Δ*bipL*	This study
MTC2069	PA14 Δ*amrZ* Δ*bipL*	This study
MTC2070	PA14 Δ*amrZ* Δ*ptsP* Δ*bipL*	This study
MTC2125	PA14 ∆*amrZ* ∆*ptsP bipL*_H268A_	This study
MTC2127	PA14 ∆*amrZ* ∆*ptsP bipL*_HKD→AAA_	This study
MTC2625	PA14 ∆*amrZ* ∆*ptsP* ∆*bipL attB*::*bipL*	This study
MTC2626	PA14 ∆*amrZ* ∆*ptsP* ∆*bipL attB*::*bipL*_S110A_	This study
MTC2632	PA14 ∆*amrZ* ∆*ptsP* ∆*bipL attB*::*bipL*_S149A_	This study
MTC2633	PA14 ∆*amrZ* ∆*ptsP* ∆*bipL attB*::*bipL*_S50A_	This study
MTC2634	PA14 ∆*amrZ* ∆*ptsP* ∆*bipL attB*::*bipL*_S98A_	This study
MTC2635	PA14 ∆*amrZ* ∆*ptsP* ∆*bipL attB*::*bipL*_S116A_	This study
MTC2636	PA14 ∆*amrZ* ∆*ptsP* ∆*bipL attB*::*bipL*_D197A_	This study
MTC2637	PA14 ∆*amrZ* ∆*ptsP* ∆*bipL attB*::*bipL*_D201A_	This study
MTC2638	PA14 *bipL-3×FLAG*	This study
MTC2639	PA14 ∆*amrZ bipL-3×FLAG*	This study
MTC2640	PA14 ∆*amrZ* ∆*ptsP bipL-3×FLAG*	This study
MTC2721	PA14 ∆*amrZ* ∆*ptsP bipL*_S149A_	This study
MTC2726	PA14 ∆*amrZ* ∆*ptsP bipL*_S149A_ *-3×FLAG*	This study

Notably, while constructing our complementation strain, we noticed that the annotated start codon of *bipL* was in fact 66 nt upstream of its transcriptional start site (at genomic coordinate 363,261) as determined by high-resolution transcriptomics (47), making it unlikely to be the true start codon. The *bipL* complementation construct we designed includes 315 nt upstream of a second AUG codon 78 nt downstream of the originally annotated start codon. Because this construct, which complements the deletion, also includes the first start codon, it does not experimentally distinguish between the two possible start codons. However, our western blotting results are consistent with the second start codon (at genomic coordinate 363,273), which, with the 3×-FLAG tag, is predicted to produce a protein of approximately 38.3 kDa vs a protein of 41.2 kDa for the first start codon (see Fig. 2C).

### Transposon mutagenesis screening

Transposon mutagenesis screening was performed as previously described (15). PA14 Δ*amrZ* Δ*ptsP* (MTC1387) was mated with *E. coli* SM10 pBT24 (MTC33), which harbors the mariner transposon. We then screened for colonies with restored wrinkled morphology in contrast to the smooth morphology of PA14 Δ*amrZ* Δ*ptsP*, indicating genes whose disruption enhances biofilm formation.

### Colony morphology assay

M6301 agar medium was used to visually assess the colony morphology of the PA14 strains. The medium consisted of 1% agar, 100  µM KH_2_PO_4_, 15.14 mM (NH_4_)_2_SO_4_, and 0.36  µM FeSO_4_·H_2_O (pH balanced to 7.0 using 10 M KOH). After autoclaving, the medium was supplemented with 0.5% glycerol, 1 mM MgSO_4_, and 0.2% casamino acids. Approximately 40 µL of the medium was poured into each petri plate and allowed to solidify at room temperature. M6301 agar plates were prepared 24–36 h prior to the experiment. Overnight cultures of PA14 cells grown with shaking at 37°C were normalized to an OD_600_ of 0.1, spotted (2 µL) onto M6301 agar plates, and grown in a 25°C incubator or on the benchtop at room temperature for 6 days before photographing. Colony photographs were captured with a Canon EOS Rebel T7i camera equipped with a Canon EF-S 35 mm macro lens against a black velvet background.

### Congo red-binding assay

To quantify extracellular matrix, colonies were scraped off the M6301 agar media using a spatula and were homogenized in 1 mL of PBS using a Cole-Parmer motorized pestle mixer (cat. no. 44468–25). Any floating bits of matrix in the resuspension mix were allowed to settle, and 100 µL of the resuspension was used to create a dilution of 1:2 to measure OD_600_ using a BioTek Synergy HT plate reader. The remaining resuspension was centrifuged at 14,500 *g* for 4 min at room temperature, and the pellet was resuspended in 40 µg/mL Congo red dye and incubated under agitation for 90 min on a GyroMixer XL (GeneMate, VWR). The resuspension was again centrifuged at 14,500 *g* for 4 min at room temperature, and 200 µL of the supernatant was used to measure unbound Congo red dye at OD_490_. Congo red concentration was calculated using a standard curve that was generated by measuring the OD_490_ of Congo red at 40, 20, 10, 5, 2, 1, and 0.5  µg mL^−1^. The amount of Congo red bound to the extracellular biofilm matrix was calculated by subtracting the unbound Congo red from the starting concentration; this number was further normalized by OD_600._ These experiments were performed with at least four biological replicates per strain or condition, and each individual data point is shown on the bar graphs. Student’s *t*-test was used to calculate significance of pairwise comparisons.

### Transcriptomic analysis

RNA sequencing was performed by growing Δ*amrZ* and Δ*amrZ* Δ*ptsP* strains in quadruplicate on solid M6301-1% agar plates for 3 days. Total RNA was isolated from homogenized colonies using the New England Biolabs Monarch Total RNA Miniprep Kit. Subsequent quality-control steps, the ribosomal RNA depletion, Illumina library preparation, and paired-end high-throughput Illumina sequencing were performed by Novogene (Beijing, China). Sequence mapping and analysis, including comparisons of transcript abundance, were performed at the Oklahoma University Health Sciences Center Laboratory for Molecular Biology and Cytometry Research using CLC software.

### Immunoblotting

Samples for immunoblots were prepared by growing cells overnight at 37°C and then normalizing to an OD_600_ of 1 in 1 mL of LB. The samples were then centrifuged at 10,000 *g* for 1 min, and the resulting pellet was resuspended in 2× or 5× SDS loading buffer at a ratio of 1:1 and boiled in a water bath for 12–15 min. The samples were run on a SDS gel for 50 min at 150 V in room temperature. The samples were then transferred from the SDS gel to a polyvinylidene fluoride (PVDF) membrane using a BioRad Trans-Blot Turbo transfer apparatus. The PVDF membrane was then blocked with Tris-buffered saline + 0.1% Tween-20 (TBST) + 5% nonfat milk powder for 1 h. For the blot shown in Fig. 2, the membrane was incubated with 1:10,000 primary antibody (Cell Signaling Technologies mouse α-FLAG primary antibody) in blocking buffer for 1 h, followed by three 5-min washes in TBST. The blot was further incubated with 1:5,000 secondary antibody (Cell Signaling Technologies HRP-conjugated horse α-mouse) in blocking solution for 1 h, again followed by three 5-min washes in TBST. For the blot shown in Fig. 4, the blocked membrane was incubated in 1:3,000 HRP-conjugated mouse monoclonal anti-FLAG M2 antibody (Sigma-Aldrich) for 3 h in the dark at 4°C followed by three 5-min washes in TBST. Bound antibodies were imaged in a BioRad ChemiDoc MP gel imaging system using the Thermo Scientific Western Pico chemiluminescence kit according to the manufacturer’s instructions.

### Lipid extraction and profiling

Bacterial strains were grown on M6301-1% agar at 25°C for 3 days, and the resulting colonies (four colonies per biological replicate; five biological replicates per strain) were scraped off the agar and homogenized in 1.6 mL PBS using a Cole-Parmer motorized pestle mixer. To the homogenized mixture, 2 mL of chloroform and 4 mL of methanol were added. The mixture was shaken well before another 2 mL of chloroform and 2 mL of water were added. After thoroughly shaking the mixture, it was centrifuged at 5,000 rpm for 5 min. Using glass pipettes, the lower, lipid-containing layer was removed. Two additional 2 mL chloroform extractions were performed, with shaking, centrifugation, and removal of the lower layer. The pooled lower layers were collected in a glass tube with a Teflon-lined screw cap. The collected mixture was cleaned by adding a small volume (0.5–1 mL) of 1M KCl, followed by gentle shaking, centrifugation, and removal of the topmost (aqueous) layer, which was discarded. A second aqueous extraction with ultrapure water was then performed. The lipids were then vacuum dried. The extracted and dried lipids were then analyzed at the Kansas State University Lipidomics Center. The raw lipidomics data are included as Data S2. To detect differences among strain pairs with or without *bipL*, the difference in the average molar percentage for each detected lipid species was calculated. The list of differences used to generate the heat map is shown in Fig. 5B.

### Phylogenetic tree construction

To build the phylogenetic tree, the FASTA sequence of BipL was used along with sequences of several genes that were identified as homologous to BipL using the NCBI BLAST website (February 2023). To incorporate diversity into the phylogenetic tree, randomly selected sequences with homology to BipL from different taxa were included. Genes were selected from different species of *Pseudomonas*, other Gammaproteobacteria, *Firmicutes*, and Archaea. Clustal Omega (EMBL) was used to align the FASTA sequences of all the selected genes. Using MEGA11, the aligned sequences were used to generate a maximum likelihood phylogenetic tree. FigTree was used to visualize and analyze the phylogenetic tree. The experimentally confirmed bacterial lipases YtpA from *Bacillus subtilis* and Rv1083 from *Mycobacterium tuberculos*is were also included.

## Data Availability

Full transcriptomic data sets comparing strains with and without *ptsP*, including the raw sequence reads for PA14 ∆*amrZ* ∆*ptsP*, have been deposited in the Gene Expression Omnibus at NCBI under accession number GSE239971. Control data sets comprising PA14 and PA14 ∆*amrZ* samples were previously published under accession number GSE226104 (48).

## SUPPLEMENTAL MATERIAL

Data Set S1 msphere.00374-23-s0001.xlsx Summary of lipid differences.

Data Set S2 msphere.00374-23-s0002.xlsx Raw lipidomics data.

Supplemental material msphere.00374-23-s0003.pdf Figures S1 to S3, Tables S1 and S2, captions for Data Sets S1 and S2, and modes of strain and plasmid construction.

ASM does not own the copyrights to Supplemental Material that may be linked to, or accessed through, an article. The authors have granted ASM a non-exclusive, world-wide license to publish the Supplemental Material files. Please contact the corresponding author directly for reuse.
