# Supplementary material for: A putative lipase affects Pseudomonas aeruginosa biofilm matrix production
Source: mSphere. 2023 Sep 27;8(5):e00374-23. doi: 10.1128/msphere.00374-23 (PMC10597414; doi:10.1128/msphere.00374-23)
Supplement: Supplemental material — Figures S1 to S3, Tables S1 and S2, captions for Data Sets S1 and S2, and modes of strain and plasmid construction. [file msphere.00374-23-s0003.pdf]

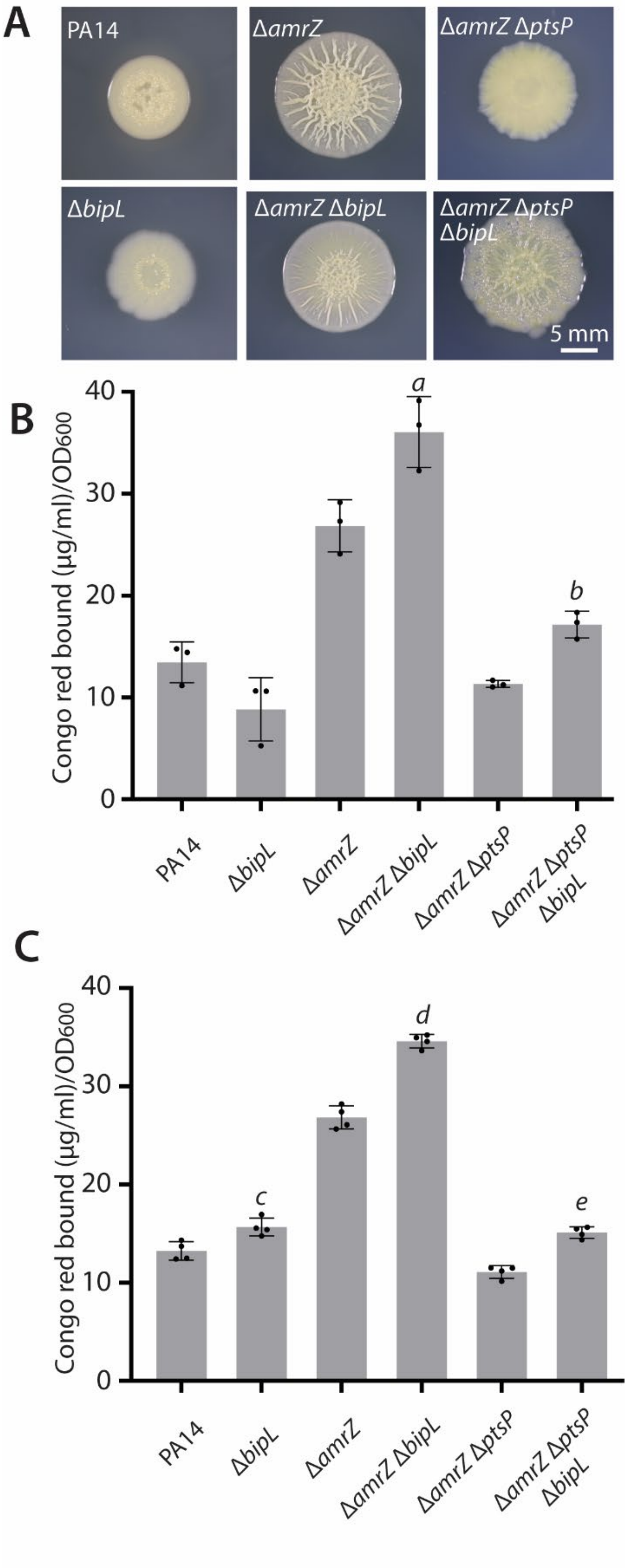

Figure S1

Figure S1. Additional representative experiments showing the impact of *bipL* deletion on colony morphology and Pel production in different strain backgrounds. **A.** Representative photographs of colony morphology of the indicated strains after 6 d of growth at 25°C on M6301 agar. **B.** Congo red binding assay of one experiment using the same strains as shown in Panel A. Bar graphs indicate the mean values of 4 biological replicates whose individual values are also shown as dots. Error bars denote standard deviation. Significance was assessed by two-tailed Student's t-testing. *a*, $P < 0.05$ vs. Δ*amrZ*; *b*, $P < 0.005$ vs. Δ*amrZ* Δ*ptsP*. The P value of the comparison between PA14 and Δ*bipL* was 0.1. **C.** Congo red binding assay of a different experiment using the same strains as shown in Panel A. Bar graphs indicate the mean values of 4 biological replicates whose individual values are also shown as dots. Error bars denote standard deviation. Significance was assessed by two-tailed Student's t-testing. *c*, $P < 0.05$ vs. PA14 (actual P value, 0.01); *d*, $P < 0.0001$ vs. Δ*amrZ*; *e*, $P < 0.0001$ vs. Δ*amrZ* Δ*ptsP*.

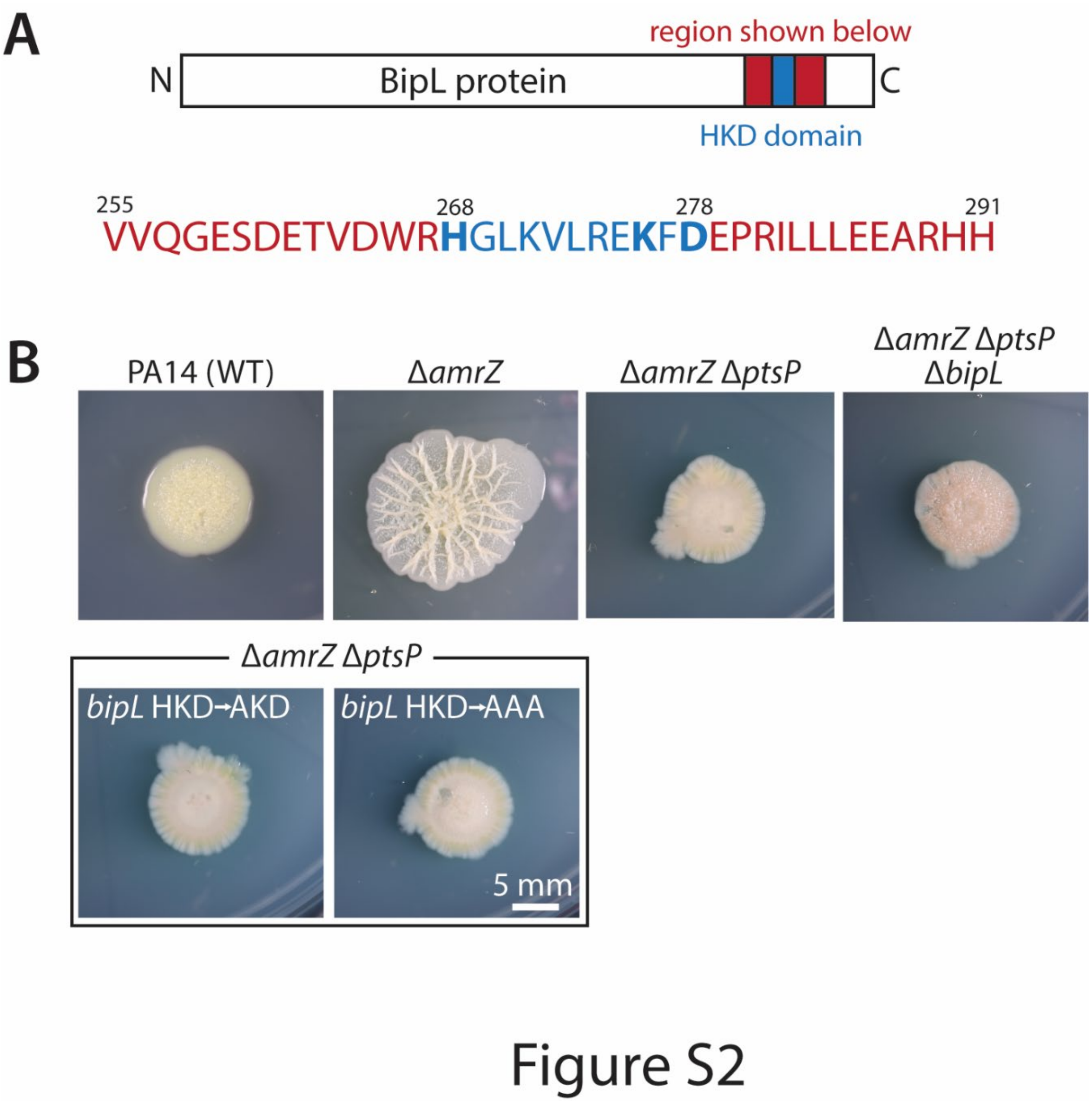

Figure S2. Impacts of amino-acid substitutions in the conserved HxxxxxxxKxD motif of BipL on colony morphology. A. Schematic of BipL protein showing the relative location of the region (red) containing the HKD motif (blue). Numbers denote the amino-acid positions. **B.** Representative photographs of colony morphology of the indicated strains after 6 d of growth at 25°C on M6301 agar.

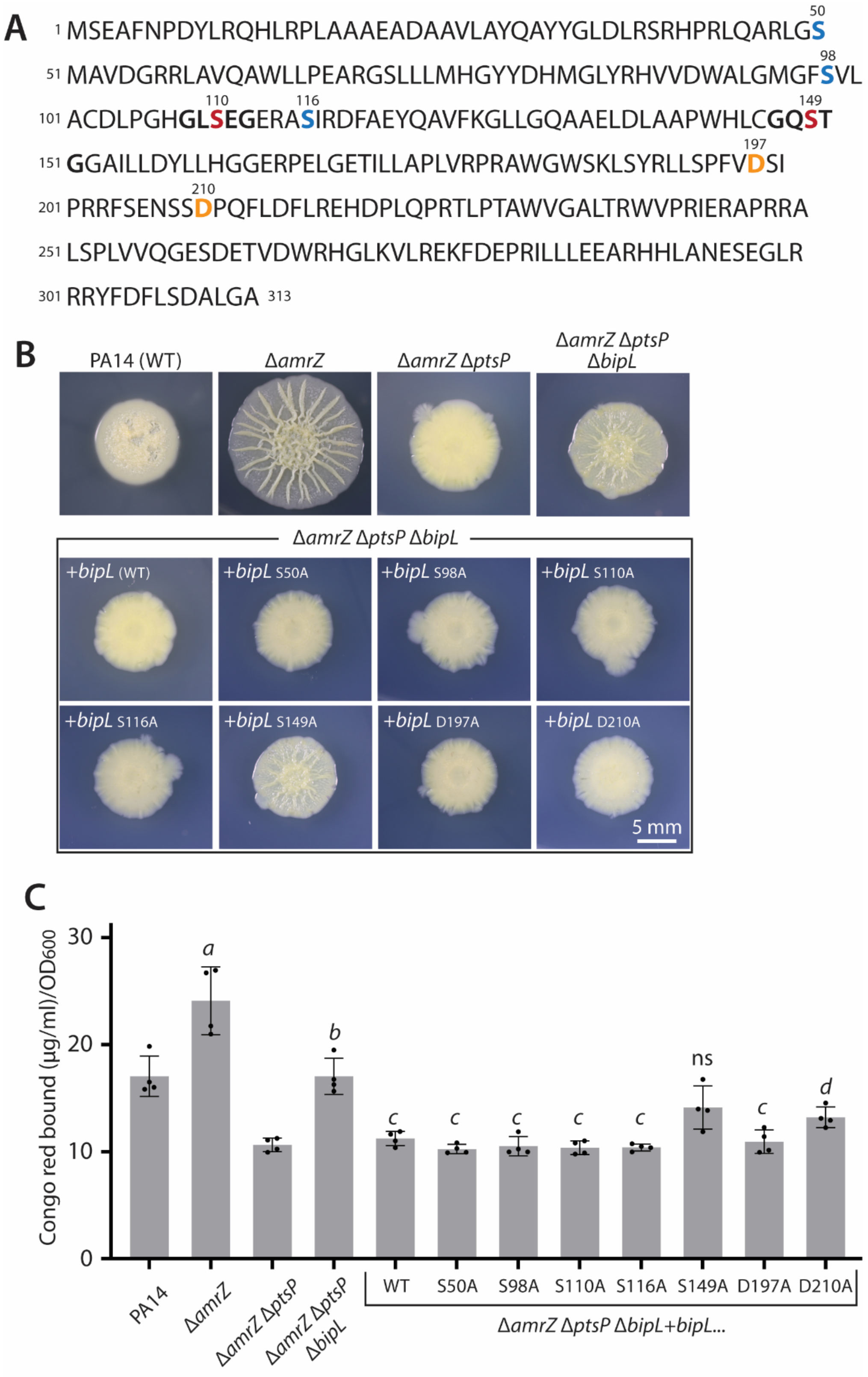
A
1 MSEAFNPDYLRQHLRPLAAAEADAAVLAYQAYYGLDLRSRHPRLQARLGS 50
51 MAVDGRRLAVQAWLLPEARGSLLLMHGYYDHMGLYRHVVDWALGMGFSVL 98
101 ACDLPGHGLSEGERASIRDFAEYQAVFKGLLGQAAELDLAAPWHLCGQST 110 116 149
151 GGAILLDYLLHGGERPELGETILLAPLVRPRAWGWSKLSYRLLSPFVDSI 197
201 PRRFSENSSDPQFLDFLREHDPLQPRTLPTAWVGALTRWVPRIERAPRRA 210
251 LSPLVVQGESDETVDWRHGLKVLREKFDEPRILLLEEARHHLANESEGLR
301 RRYFDFLSDALGA 313
B
PA14 (WT)
ΔamrZ
ΔamrZ ΔptsP
ΔamrZ ΔptsP ΔbipL
ΔamrZ ΔptsP ΔbipL
+bipL (WT)
+bipL S50A
+bipL S98A
+bipL S110A
+bipL S116A
+bipL S149A
+bipL D197A
+bipL D210A
5 mm
C
Congo red bound (μg/ml)/OD600
0
10
20
30
a
b
ns
c
d
PA14
ΔamrZ
ΔamrZ ΔptsP
ΔamrZ ΔptsP ΔbipL
WT
S50A
S98A
S110A
S116A
S149A
D197A
D210A
ΔamrZ ΔptsP ΔbipL+bipL...

Figure S3

Figure S3. Impacts of Ser and Asp amino-acid substitutions in BipL on colony morphology and Pel production. A. Sequence of BipL protein showing the locations of Ser residues (bold, blue or red) and Asp residues (bold, orange) tested by Ala substitution. GXSXG lipase signature motifs are shown in boldface black type, with their putative catalytic Ser residues shown in red. Numbers denote the amino-acid positions. **B.** Representative photographs of colony morphology of the indicated strains after 6 d of growth at 25°C on M6301 agar. **C.** Congo red binding assay of one experiment using the same strains as shown in Panel A. Bar graphs indicate the mean values of 4 biological replicates whose individual values are also shown as dots. Error bars denote standard deviation. Significance was assessed by two-tailed Student's t-testing. *a*, P < 0.01 vs. PA14; *b*, P < 0.001 vs. Δ*amrZ* Δ*ptsP*, *c*, P < 0.001 vs. Δ*amrZ* Δ*ptsP* Δ*bipL*; *d*, P < 0.01 vs. both Δ*amrZ* Δ*ptsP* and Δ*amrZ* Δ*ptsP* Δ*bipL*; ns, P > 0.05 vs. Δ*amrZ* Δ*ptsP* Δ*bipL*. Note that the images of and Congo red binding data for the control strains PA14, Δ*amrZ*, Δ*amrZ* Δ*ptsP*, Δ*amrZ* Δ*ptsP* Δ*bipL*, Δ*amrZ* Δ*ptsP* Δ*bipL attB*::*bipL* (WT), Δ*amrZ* Δ*ptsP* Δ*bipL attB*::$bipL_{S110A}$, and Δ*amrZ* Δ*ptsP* Δ*bipL attB*::$bipL_{S149A}$ are identical to those shown in Figs. 1 and 4 of the main text and were taken from the same representative experiment that included all the strains shown in Figs. 1 and 4.

Table S1. Plasmids used in this study.

Plasmid	Description	Source or reference
pCTX-1	mini-CTX-1, integrative tet^R plasmid for *P. aeruginosa.*	(1)
pEXG2	Integrating suicide plasmid for *P. aeruginosa*, gent^R, with sucrose counterselection.	(2)
pEXG2-Δ*04030*	EXG2 containing flanking sequences of *04030* (~600 bp).	This study
pEXG2-04030_{S149A}	EXG2 containing flanking sequences of 04030_{S149A} (~600 bp).	This study
pEXG2-04030_{S110A}	EXG2 containing flanking sequences of 04030_{S110A} (~600 bp).	This study
pCTX-1-*04030*	CTX-1 containing promoter (~318 bp) and coding sequence of *04030* gene.	This study
pCTX-1-04030_{S149A}	CTX-1 containing promoter (~318 bp) and coding sequence of *04030* gene encoding substitution of serine 149 with alanine.	This study
pCTX-1-04030_{S110A}	CTX-1 containing promoter (~318 bp) and coding sequence of *04030* gene encoding substitution of serine 110 with alanine.	This study
pCTX-1-04030_{S50A}	CTX-1 containing promoter (~318 bp) and coding sequence of *04030* gene encoding substitution of serine 50 with alanine.	This study
pCTX-1-04030_{S98A}	CTX-1 containing promoter (~318 bp) and coding sequence of *04030* gene encoding substitution of serine 98 with alanine.	This study
pCTX-1-04030_{S116A}	CTX-1 containing promoter (~318 bp) and coding sequence of *04030* gene encoding substitution of serine 116 with alanine.	This study
pCTX-1-04030_{D197A}	CTX-1 containing promoter (~318 bp) and coding sequence of *04030* gene encoding substitution of aspartic acid 197 with alanine.	This study
pCTX-1-04030_{D201A}	CTX-1 containing promoter (~318 bp) and coding sequence of *04030* gene encoding substitution of aspartic acid 201 with alanine.	This study
pEXG2-*04030_FLAG*	EXG2 containing flanking sequences of *04030* (~600 bp) and coding sequence of *04030* gene with a C-terminal *3X-FLAG* tag.	This study
pEXG2-04030_{S149A}*_FLAG*	EXG2 containing flanking sequences of 04030_{S149A} (~600 bp) and coding sequence of *04030* gene with a C-terminal *3X-FLAG* tag.	This study

Table S2. Primers used in this study.

**Primer Name or Number**	**Sequence (5'-3')**
Rnd1-ARB1	GGCCACGCGTCGACTAGTACNNNNNNNNNNAGAG
Rnd1-ARB2	GGCCACGCGTCGACTAGTACNNNNNNNNNNACGCC
Rnd1-ARB3	GGCCACGCGTCGACTAGTACNNNNNNNNNNGATAT
Rnd1-TnM20	TATAATGTGTGGAATTGTGAGCGG
Rnd2-ARB	GGCCACGCGTCGACTAGTAC
Rnd2-TnM20	ACAGGAAACAGGACTCTAGAGG
BT20TnMseq	CACCCAGCTTTCTTGTACAC
834 / EXG2_Eco_PA0308_Up_F	**TGCGCACCCGTGGAAATTAATTAAGGTACCGAATT**<br>**C** CGGTGTTGAGCCAGGTCAGC
835 / PA0308_up_R	**CAGCGCGTCG** CGCAGGTCCAGGCCGTAG
836 / PA0308_down_F	**TGGACCTGCG** CGACGCGCTGGGGGCCTG
837 / EXG2_Hind_PA0308_down_R	**TTATACGAGCCGGAAGCATAAATGTAAAGCAAGCT**<br>**T** CAACAGCAATCGCCGCTTCGC
1056 / CTX-1_Eco_04030_F	**TCTAGAACTAGTGGATCCCCCGGGCTGCAG**<br>**GAATTC** GGGTCCCAGAAATGCACCAGGCGG
1057 / CTX-1_Hind_04030_R	**CCCCCTCGAGGTCGACGGTATCGAT AAGCTT**<br>CCTGGAATTCAGGCCCCCAGCGC
1092 / 04030_S50A_Q5_F	GCGGCTCGGCGCCATGGCGGTCG
1093 / 04030_S50A_Q5_R	GCTTGCAGGCGCGGATGG
1096 / 04030_S98A_Q5_F	CATGGGCTTCGCCGTGCTCGCCTGC
1097 / 04030_S98A_Q5_R	CCCAGCGCCCAGTCGACC
1098 / 04030_S116A_Q5_F	CGAGCGGGCCGCCATCCGCGACTTC
1099 / 04030_S116A_Q5_R	CCTTCGGCCAGGCCATGC
1100 / 04030_S149A_Q5_F	CTGCGGACAAGCCACCGGTGGGGCGATCCTGC
1101 / 04030_S149A_Q5_R	AGGTG<br>CCAGGGCGCCGCC
1102 / 04030_D197A_Q5_F	CCCCTTCGTCGCCTCGATCCCGC
1103 / 04030_D197A_Q5_R	CTCAGCAGGCGGTAGCTG
1104 / 04030_D210A_Q5_F	GAACTCCAGCGCCCCGCAGTTCCTCGACTTC
1105 / 04030_D210A_Q5_R	TCCGAGAAGCGCCGCGGG
1000 / EXG2_Eco_04030_S110A_up_F	**TGCGCACCCGTGGAAATTAATTAAGGTACC**<br>**GAATTC** GGGTCCCAGAAATGCACCAGGCGG
1001 / 04030_S110A_up_R	TCGCCTTCGG**C**CAGGCCATGCCCCGG
1002 / 04030_S110A_down_F	GGGGCATG**G**CCTGGCCGAAGGCGA
1003 / EXG2_Hind_04030_down_R	**TTATACGAGCCGGAAGCATAAATGTAAAGC**<br>**AAGCTT** CCTGGAATTCAGGCCCCCAGCGC
1235 / EXG2_Eco_PA04030_up_F	**TGCGCACCCGTGGAAATTAATTAAGGTACCGAATT**<br>**C** AGGCGAGCGGGCCAGCATC
1236 / 04030_Flag_up_R	**CTTTATCGTCGTCATCTTTG**TAATCGGAGCCGCCGGA<br>GCCGCCGGCCCCCAGCGCGTCGC
1237 / 04030_Flag_down_F	CAAAGATGACGACGATAAAGATTACAAAGATGACGAC<br>GATAAAGATTACAAAGATGACGATAAA<br>**TGAATTCCAGGCCCCGCGGC**
1238 / EXG2_Hind_PA04030_down_R	**TTATACGAGCCGGAAGCATAAATGTAAAGCAAGCT**<br>**T** CAACAGCAATCGCCGCTTCGC

NB: The listed primer sequences may include 5' overlaps for isothermal assembly and/or stitch PCR (in boldface type). The 3' end is complementary to the target genomic sequence.

Supplemental Dataset S1 | List of differences in lipid species

This Excel spreadsheet lists in each row the mass, chemical formula, and lipid species name of selected lipids from the lipidomics data. Column D shows the difference in the average molar percentage of each lipid species between PA14 and PA14 Δ*04030* (*bipL*), and column E shows the difference in the average molar percentage of each lipid species between PA14 Δ*amrZ* Δ*ptsP* and PA14 Δ*amrZ* Δ*ptsP* Δ*04030* (*bipL*). Columns F and G show the P-values for Student's t-test between the individual biological replicate (5 per sample) distributions for each lipid species in the respective comparisons.

Supplemental Dataset S2 | Raw lipidomics data

This Excel spreadsheet contains the full lipidomics dataset that was received from the Kansas State Lipidomics center, including the mass spectrometry parameters, background subtraction data, lipid amounts, control quantities, molar percentages, averages, and summaries. Each of the data types are listed on labeled tabs, and additional annotation is included in the spreadsheet.

Modes of strain construction

Pseudomonas aeruginosa strains

CSS198

This strain was isolated from the transposon mutagenesis screening experiment. The location of the transposon insertion was verified by sequencing using transposon specific primers as elaborated under the experimental procedures.

MTC2070 (CSS358)

CSS 15 was mated with MTC 2050. 50 µl of MTC 2050, which is the donor *E.coli* strain is first spot dried on LB plate and subsequently 100 µl of MTC 1, the recipient strain is spot dried on top of it. The plate was incubated at 37°C overnight and the mated colony was scraped up using a sterile inoculation loop and resuspended in 500 µl of LB and vortexed thoroughly. 100 µl of the resuspension mix was spread on LB agar plates supplemented with 25 µg/µl of irgasan and 75 µg/µl of gentamycin and incubated overnight at 37°C. This step selects for *Pseudomonas aeruginosa* transformants that acquired the plasmid pEXG2-Δ*04030* by homologous recombination. 6-8 colonies of the above *P. aeruginosa* transformants were picked and inoculated in plain LB and incubated at 37°C for the second crossover to happen. 100 µl aliquots of the culture was spread on LB agar supplemented with 6% sucrose to select for colonies that lost the plasmid backbone after the second crossover. 20-30 sucrose resistant colonies were patched on plain LB agar and LB agar with 20 µg/µl gentamycin to select for gentamycin sensitive colonies to further validate the loss of the plasmid. 2 of the gentamycin sensitive colonies were streaked on LB agar and were checked by PCR to verify the deletion of the gene of interest. Once verified, the strain was frozen at -80°C in 50% glycerol.

MTC2032

Constructed like CSS 358, but MTC 2016 was mated with MTC 1387.

MTC2068 (CSS362)

Constructed like CSS 358, but MTC 2050 was mated with MTC 1.

MTC2069 (CSS361)

Constructed like CSS 358, but MTC 2050 was mated with MTC 590.

MTC2625 (CSS964)

CSS 358 was mated with MTC 2153 as described above for CSS 358. 25-100 µl aliquots were spread on LB agar supplemented with 25 µg/µl of irgasan and 75 µg/µl of tetracycline to select for PA14 transformants with the integrated plasmid. 2 isolated colonies were selected and restreaked on LB agar with 25 µg/µl of tetracycline and incubated at 37°C overnight for isolated colonies. Liquid cultures were made using those isolated colonies and frozen at -80°C in 50% glycerol.

MTC2626 (CSS1047)

Constructed like CSS 964, but CSS 358 was mated with MTC 2155.

MTC2632 (CSS885)

Constructed like CSS 964, but CSS 358 was mated with CSS 873.

MTC2721 (CSS1131)

Constructed like CSS 358, but CSS 15 was mated with CSS 1010. The point mutation was verified by sequencing.

MTC2638 (CSS1174)

Constructed like CSS 358, but MTC 1 was mated with CSS 1011. The presence of the *3X-FLAG* was verified by sequencing.

MTC2639 (CSS1069)

Constructed like CSS 358, but MTC 590 was mated with CSS 1011. The presence of the *3X-FLAG* was verified by sequencing.

MTC2640 (CSS1070)

Constructed like CSS 358, but CSS 15 was mated with CSS 1011. The presence of the *3X-FLAG* was verified by sequencing.

MTC2633 (CSS875)

Constructed like CSS 964, but CSS 358 was mated with CSS 858.

MTC2634 (CSS876)

Constructed like CSS 964, but CSS 358 was mated with CSS 862.

MTC2635 (CSS866)

Constructed like CSS 964, but CSS 358 was mated with CSS 857.

MTC2636 (CSS865)

Constructed like CSS 964, but CSS 358 was mated with CSS 860.

MTC2637 (CSS877)

Constructed like CSS 964, but CSS 358 was mated with CSS 874.

MTC2721 (CSS1964)

Constructed like CSS 358, but CSS 1926 was mated with MTC 2721. The point mutation was verified by sequencing.

Modes of plasmid construction

All plasmids constructed in this study were assembled from purified PCR products and restriction enzyme-cleaved plasmid backbones by using isothermal assembly (Gibson *et.al.*, 2009). The insert sequences of all plasmids were verified via Sanger sequencing before they were used to create new strains.

pEXG2-Δ*04030*

The upstream and downstream flanking regions (~600 bp each) of *04030* was amplified from PA14 genomic DNA using the primer pairs 834/835 and 836/837 respectively. A fragment containing the *04030*

gene deletion was generated by stitch PCR using the initial fragments as self-priming templates with primers 834 and 837. The resulting deletion fragment was assembled into EcoRI/HindIII-cleaved pEXG2.

pCTX-1-*04030*

The 04030 gene was amplified from the PA14 genomic DNA using the primers 1056 and 1057. The resulting fragment was assembled into EcoRI/HindIII-cleaved mini-CTX-1.

pEXG2-*04030_{S110A}*

The version of 04030 encoding the S110A substitution was generated via mutagenic PCR using primers 1000/1001 for the upstream flank and 1002/1003 for the downstream flank. The full fragment was generated by stitch PCR using the initial fragments as self-priming templates with primers 1000 and 1003. The resulting *04030_{S110A}* fragment was assembled into EcoRI/HindIII-cleaved pEXG2.

pCTX-1-*04030_{S110A}*

The *04030_{S110A}* gene was amplified from pEXG2-*04030_{S110A}* using the primers 1056 and 1057. The resulting fragment was assembled into EcoRI/HindIII-cleaved mini-CTX-1.

pCTX-1-*04030_{S149A}*

The plasmid was amplified using the Q5 Site-Directed Mutagenesis Kit Protocol (NEB) using pCTX-1-*04030* as the template plasmid and 1100 and 1101 as the respective mutagenic primers.

pCTX-1-*04030_{S50A}*

The plasmid was amplified using the Q5 Site-Directed Mutagenesis Kit Protocol (NEB) using pCTX-1-*04030* as the template plasmid and 1092 and 1093 as the respective mutagenic primers.

pCTX-1-*04030_{S98A}*

The plasmid was amplified using the Q5 Site-Directed Mutagenesis Kit Protocol (NEB) using pCTX-1-*04030* as the template plasmid and 1096 and 1097 as the respective mutagenic primers.

pCTX-1-*04030_{S116A}*

The plasmid was amplified using the Q5 Site-Directed Mutagenesis Kit Protocol (NEB) using pCTX-1-*04030* as the template plasmid and 1098 and 1099 as the respective mutagenic primers.

pCTX-1-*04030_{D197A}*

The plasmid was amplified using the Q5 Site-Directed Mutagenesis Kit Protocol (NEB) using pCTX-1-*04030* as the template plasmid and 1102 and 1103 as the respective mutagenic primers.

pCTX-1-*04030_{D201A}*

The plasmid was amplified using the Q5 Site-Directed Mutagenesis Kit Protocol (NEB) using pCTX-1-*04030* as the template plasmid and 1104 and 1105 as the respective mutagenic primers.

pEXG2-*04030_FLAG*

The upstream and downstream flanks to construct *04030_3X-FLAG* were amplified from PA14 genomic DNA using the primer pairs and 1235/1236 and 1237/1238 respectively. A fragment containing the *04030_3X-FLAG* was then generated by stitch PCR using the initial fragments as self-priming templates with primers 1235 and 1238. The resulting deletion fragment was assembled into EcoRI/HindIII-cleaved pEXG2.

pEXG2-*04030_{S149A}_FLAG*

The upstream and downstream flanks to construct *04030_3X-FLAG* were amplified from PA14 $bipL_{S149A}$ genomic DNA using the primer pairs and 1235/1236 and 1237/1238 respectively. A fragment containing the 04030_{S149A}*_3X-FLAG* was then generated by stitch PCR using the initial fragments as self-priming templates with primers 1235 and 1238. The resulting deletion fragment was assembled into EcoRI/HindIII-cleaved pEXG2.
